# Synthesis and characterization of Co-MOF@Ag_2_O nanocomposite and its application as a nano-organic catalyst for one-pot synthesis of pyrazolopyranopyrimidines

**DOI:** 10.1038/s41598-023-44667-6

**Published:** 2023-10-15

**Authors:** Ghader Hootifard, Enayatollah Sheikhhosseini, Sayed Ali Ahmadi, Mahdieh Yahyazadehfar

**Affiliations:** Department of Chemistry, Kerman Branch, Islamic Azad University, Kerman, Iran

**Keywords:** Chemistry, Materials science, Nanoscience and technology

## Abstract

In this study, a Co-MOF was synthesized via a co-precipitation procedure and then used as support for stabilizing Ag ions and producing Co-MOF@Ag_2_O nanocomposite by microwave irradiation. The characterization of synthesized Co-MOF@Ag_2_O nanocomposite was performed by using different techniques such as field emission scanning electron microscopy (FE-SEM), energy dispersive X-ray (EDX) analyses, X-ray diffraction (XRD), Thermogravimetric analysis (TGA), Brunauer–Emmett–Teller (BET) and Fourier-transform infrared (FT-IR). The prepared Co-MOF@Ag_2_O nanocomposite was applied as a heterogeneous nano-catalyst in the synthesis of pyrazolopyranopyrimidines in water at 50 °C via the one-pot multicomponent reaction of ethyl acetoacetate, hydrazine hydrate, aromatic aldehydes and barbituric acid derivatives. Through this straightforward and effective protocol, different tricyclic fused pyrazolopyranopyrimidines were synthesized at high yields, and short reaction times, through an uncomplicated work-up process with no by-product. The Co-MOF@Ag_2_O nanocomposite has been effectively recycled for four consecutive cycles without appreciable loss in its activity. Cost-effectiveness, no need for column chromatography, mild conditions, catalyst recyclability, and eco-friendly nature make it a promising candidate compared to other methods.

## Introduction

The unique characteristics of MOFs such as high surface area, considerable and tunable porosity, tunable pore size and shape, high physical and chemical stability, the presence of active binding sites within the framework, amphiphilic microenvironment, diverse structures, large pore size, nanometer-scale size, and biodegradability^1–3^ have caused their importance and application in various industrial and biomedical fields, in which the following can be mentioned: generation of MOF-based metal and metal oxide nanocomposites as heterogeneous catalysts or as catalyst supports/precursors in organic reactions, catalyze a vast range of transformations from organic reaction to photocatalysis^4^, as stationary phases for liquid and gas chromatographies and as the efficient adsorption materials in sample pretreatment^5^, application as agents' gas and energy storage, heat transformation, imaging, molecular separation, sensing, biosensors, contrast agents for magnetic resonance imaging and etc^6,7^.

Among various metal oxides, silver oxide (Ag_2_O) is the best candidate for the synthesis of heterogeneous catalysts due to its proper band gap energy (1.2 eV). Silver oxide nanoparticles are p-type semiconductors with vast applications in the field of catalysis, sensors, fuel cells, and photovoltaic cells^8–11^. Many physical and chemical properties such as luminescence, conductivity, and catalytic activity depend on the size of nanoscale materials. Numerous methods have been developed for the preparation of Ag_2_O among which, photosensitized reduction, simple chemical method, and electrochemical synthesis can be mentioned^12–14^.

Multi-component reactions (MCR) are convergent one-pot processes that enable three or more components to connecting a series of chemical reactions that can lead to enable easy, efficient, automated, and high-throughput synthesis of a wide range of complex natural, heterocyclic, and pharmaceutical structures by combining three or more substrates^15,16^. Solvent, inter-coordination between the reactants, and catalyst are crucial for the success of MCRs in the sequence of the reaction^17^. The most remarkable attribute of MCRs is that almost all the parts and features of the reactants can be found in the newly formed product indicative of low by-product formation making them wonders of reaction design and efficiency^15^. The development of new MCRs, and improvement of known multi-component reactions are due to their outstanding advantages such as simple operation, high selectivity, high efficiency, high atom economy, reducing the generate of by-products and waste, cost-effectiveness, Saving the chemical materials, time, energy and solvent, eco-friendly nature^18–24^.

**Figure 1:**
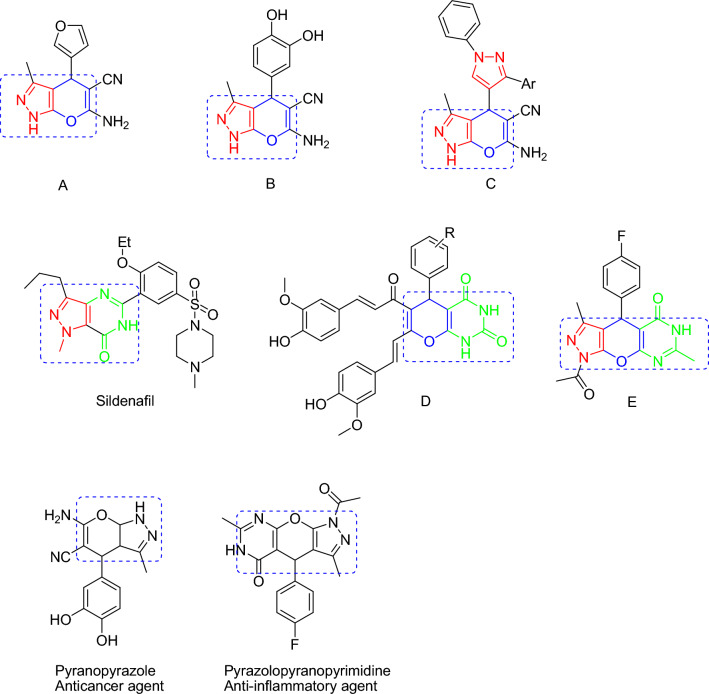
Structure of bio-active pyranopyrazole, pyranopyrimidine, and pyrazolopyranopyrimidine.

Fused heterocyclic rings can be abundantly found in nature. One of the most noticeable effects is related to the presence of two or more different moieties in one molecule due to the possibility of possessing the features of all moieties and enhancing pharmacological functions. Recently, considerable developments have been made in the design of new polycyclic heterocycles and fused heterocyclic through combining different motifs of diverse structures. Biological science essentially needs to develop new techniques to synthesize fused heterocyclic compounds^25^. The extensive applications of fused heterocycles comprising pyrimidine, pyran, and pyrazole fragments in pharmaceutics, medicine, and material sciences, along with their biodegradability have raised their popularity^26^. The pyrimidine nucleus significantly contributes to several pharmacological agents with extensive biological functions^27^. Similarly, the pyran nucleus is an important scaffold in different natural production^28^. Moreover, Pyrazole is a necessary heterocycle analog with a critical role in different pharmaceutical and agrochemical processes^29^. Pyrazolopyranopyrimidine and its derivatives are widely used in biological applications. For instance, pyranopyrazole **A** serves as a molluscicidal agent^30^. Pyranopyrazole B is considered a Chk1 inhibitor^31^ while pyranopyrazole **C** is an antimicrobial agent^32^. Another instance is the pyrazolopyrimidine core in the popular medicine called sildenafil. Figure 1 shows that the Pyranopyrimidine unit in **D** performs the role of an anti-diabetic agent^33^, while pyrazolopyranopyrimidine **E** can provide anti-inflammation effects^34^.

As polycyclic heterocycles, pyrazolopyranopyrimidines comprise pyrimidine, pyran, and pyrazole moieties. The pyranopyrimidine fragment has several nature-abundant biological functions such as tyrosine kinase inhibition^35^, anticancer features^36^, antiallergy^37^, antitubercular, antimicrobial, antihypertension^38^, antifolates^39^, antileishmanial^40^, anti-inflammation^41^, antibronchitis^42^, hypoglycaemic^35^, anticonvulsants^43^, antidepressants^37^, analgesics^41^, diuretics^44^, hepatoprotection^45^, and calcium channel antagonists^46^. Studies have also shown the critical role of the pyranopyrazole fragment in pharmaceutical and agrochemical industries^30^ while representing interesting patterns in pharmaceutics and valuable contributions to various biological functions of molecules.

The synthesis of pyrazolopyranopyrimidines mainly involves the four-component condensation of aryl aldehydes, barbituric acid derivatives, ethyl acetoacetate, and hydrazine hydrate. As shown by previous studies, pyrazolopyranopyrimidines can be prepared through the application of different catalytic agents, including SDS^47^, DABCO^48^, ChCl:Urea^49^, Halloysite clay nanotubes^50^, Magnetized Water^51^, Nano-ZnO^52^, TiO_2_ nanowire^53^, Meglumine^54^, cellulose-based nanocomposite^55^, silica-bonded (SB DBU^+^Cl^−^), (SB-DABCO^+^Cl^−^), (NSB-DBU^+^Cl^−^)^56^, MNS-ionic liquid^57^, OMWC nanotubes^58^, SBA-PR-SO_3_H^59^, oleic acid^60^, and Cu-IMS nanoparticles^61^.

Each of these catalytic systems has simultaneously their own advantages and disadvantages for catalyzing this reaction. Some of them suffer from disadvantages such as long reaction time^58,61^ and hard preparation process to prepare the catalyst^50^. Hence, there is still a need for more attempts to design and explore new and more efficient catalytic systems with tailored functional groups, that address green and sustainable chemistry principles. In this regard, the current study applied Co-MOF@Ag_2_O in a green medium to develop a safe protocol for the synthesis of pyrazolopyranopyrimidine derivatives. The novelty of this precedure is the synthesis of a new and recyclable Co-MOF@Ag_2_O nano-catalyst and using it in preparation of the heterocyclic synthesis in organic reaction efficiently.

## Experimental section

### Chemicals and reagents

Silver nitrate, cobalt(II) nitrate, dimethylformamide, 2,6-pyridinedicarboxylic acid, ethanol, sodium hydroxide, Hydrazine hydrate, ethyl acetoacetate, aryl aldehydes, and barbituric acid derivatives were purchased from Sigma-Aldrich. The chemicals were used without further purification.

### Material characterization

Melting point (m.p.) measurement was performed by an open capillary tube method using an electrothermal 9200 apparatus. Reactions were monitored by TLC. FT-IR spectra were obtained as potassium bromide pellets in the range of 400–4000 cm^−1^ on a Bruker FT-IR Tensor 27 spectrophotometer. Nuclear magnetic resonance (NMR) spectra were obtained on a Bruker DRX-400 Avance instrument (400 MHz for ^1^H) with DMSO-*d*_*6*_ as solvent. Chemical shifts were expressed in parts per million (ppm), and coupling constant (*J*) was reported in hertz (Hz). All the known compounds were identified by comparison of their melting points with the corresponding values in the literature. A Philips analytical PC-APD X-ray diffractometer operating with Kα radiation (α_2_, λ_2_ = 1.54439 Å) and graphite mono-chromatic Cu radiation (α_1_, λ_1_ = 1.54056 Å) was employed for the X-ray powder diffraction (XRD) analysis to assess the crystalline condition of the product. Then, scanning electron microscopy equipped with an energy-dispersive X-ray spectroscope (KYKY & EM 3200) was utilized for morphological investigation of the Co-MOF@Ag_2_O nanocomposite. Thermal behavior was analyzed in N_2_ from room temperature to 350 °C using a STA-1500 thermoanalyzer. N_2_ adsorption–desorption isotherms (BET) were measured on a TriStar II Plus surface area and porosity analyzer at 77 K.

### Synthesis of Co-MOF@Ag_2_O nanocomposite

#### Synthesis of Ag_2_O nanoparticles

Synthesis of Ag_2_O nanoparticles involved the dissolution of 0.005 mmol of silver nitrate in 80 mL deionized water, followed by the dropwise addition of 20 mL of 0.025 NaOH which immediately led to precipitation. Finally, the products were separated and rinsed with distilled water for 30 min, and dried in an oven at 130 °C^62^.

#### Synthesis of Co-MOF

8.186 mmol cobalt(II) nitrate was added to the solution of 2,6-pyridinedicarboxylic acid-linker (3.101 g) in the least volume of dimethylformamide, followed by 8 h of stirring at 100 °C. The resulting Co-MOF precipitates were finally collected and the residual raw materials were eliminated by washing with dimethylformamide and ethanol three times followed by 12 h of drying at 100 °C^63,64^.

#### Synthesis of Co-MOF@Ag_2_O nanocomposite

Synthesis of Co-MOF@Ag_2_O nanocomposite involved dispersion of a 0.6 g powder of the dried Co-MOF in deionized water followed by adding 0.179 g (1 mmol) of Ag_2_O under stirring at 80 °C for 10 min to reach a homogeneous solution. The final powder mixture was transferred to a glassy vial for microwave irradiation. (300 W) for 70 min. The raw materials were eliminated by washing with acetic acid. The dried powder was calcined in a furnace at 170 °C.

#### General procedure for the synthesis of pyrazolopyranopyrimidines

Hydrazine hydrate (1.0 mmol), ethyl acetoacetate (1.0 mmol), aldehyde (1.0 mmol), and barbituric acid (1.0 mmol) were added to a 50 mL round-bottom flask containing Co-MOF@Ag_2_O (20 wt%, 0.030 g) and water (5.0 mL) and stirred at 50 °C. The progress of the reaction was monitored by thin- layer chromatography. After completion of the reaction, the mixture was filtered, then the resulting precipitate was dissolved in hot ethanol and the Co-MOF@Ag_2_O nano-catalyst was separated by filtration. The solution was concentrated by solvent evaporation and the resulting precipitate was washed with water to achieve pure products. The catalyst was recovered by separation followed by washing with water and acetone (5 mL) twice to be reused after drying in an oven at 65 °C.

#### Selected spectral data

*4-(2-hydroxynaphthalen-1-yl)-3-methyl-4,8-dihydropyrazolo[4',3':5,6]pyrano[2,3-d]pyrimidine-5,7(1H,6H)-dione (5r)*: Yield 90%, m.p: 220–221 °C; ^1^H NMR (400 MHz, DMSO-*d*_*6*_*,* ppm): δ 2.62 (s, 3H, CH_3_), 5.35 (s, 1H, CH), 7.38 (d, 1H, *J*_1_ = 9.2 Hz), 7.51–7.55 (m, 2H, 1H-Ar, 1H-NH), 7.62 (td, 1H, *J*_1_ = 15.6 Hz, *J*_2_ = 1.2 Hz, H-Ar), 7.70 (td, 1H, *J*_1_ = 15.6 Hz, *J*_2_ = 1.2 Hz, H-Ar), 7.97 (d, *J* = 8.8 Hz, 1H, H-Ar), 8.11 (d, *J* = 9.2 Hz, 1H, H-Ar), 8.74 (s, 1H, OH), 10.09 (s, 1H, NH), 12.98 (s, 1H, NH) ppm; ^13^C NMR (100 Hz, DMSO-*d*_6_): δ 10.0, 23.3, 87.9, 108.4, 116.4, 118.9, 121.8, 123.8, 124.9, 127.0, 128.0, 129.0, 130.7, 131.0, 132.3, 134.7, 160.0, 161.3 ppm.

## Results and discussion

### Characterization of Co-MOF@Ag_2_O nanocomposite

**Figure 2:**
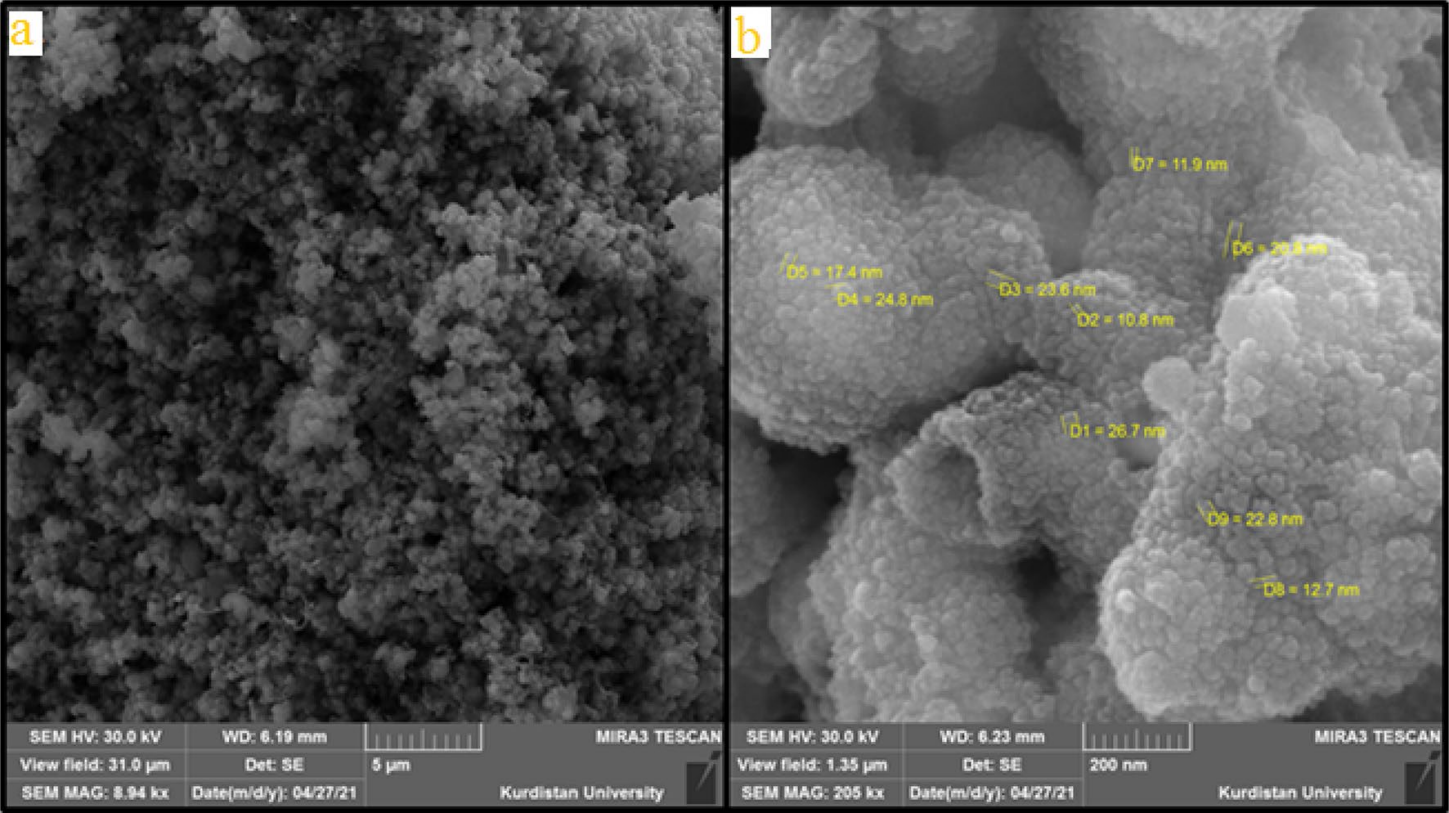
(**a**) FE-SEM image and (**b**) high-resolution FE-SEM image of the Co-MOF@Ag_2_O nanocomposite.

Scanning electron microscopy (SEM) image shows (Fig. 2) the morphological features of Co-MOF@Ag_2_O nanocomposite. This image shows the formation of aggregated spherical Co-MOF@Ag_2_O nanocomposite particles with a size range of 11 to 30 nm.

**Figure 3:**
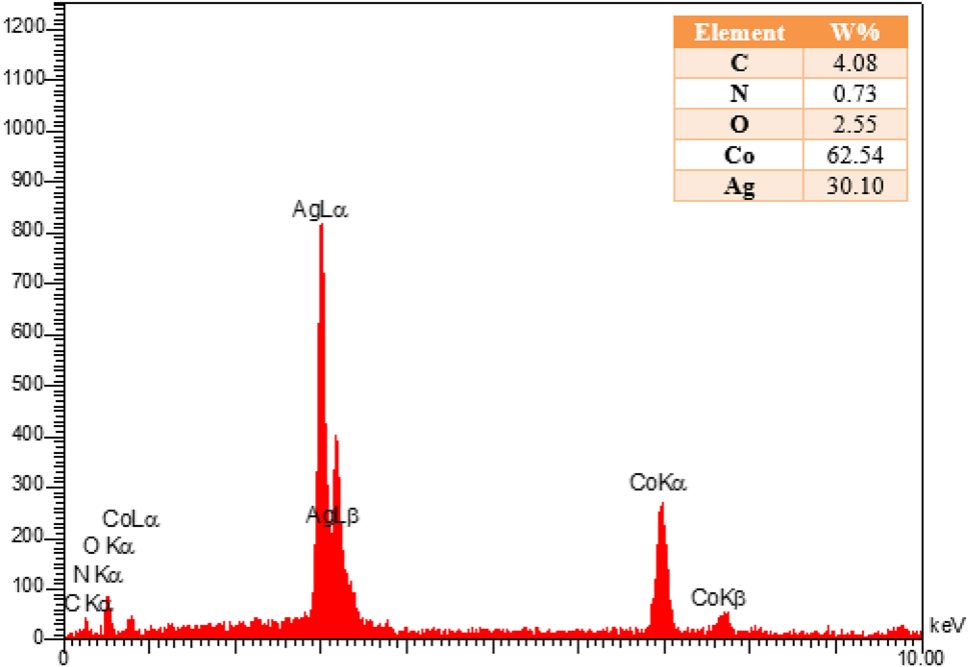
EDX spectra of Co-MOF@Ag_2_O nanocomposite.

Energy-dispersive X-ray spectroscopy (EDX) results show the elemental analysis of Co-MOF@Ag_2_O nanocomposite (Fig. 3). The strong signal at 3 keV can be assigned to silver while some weak signals can be seen related to Co, N, O, and C. The major emission energy at 3 keV indicates the presence of silver.

**Figure 4:**
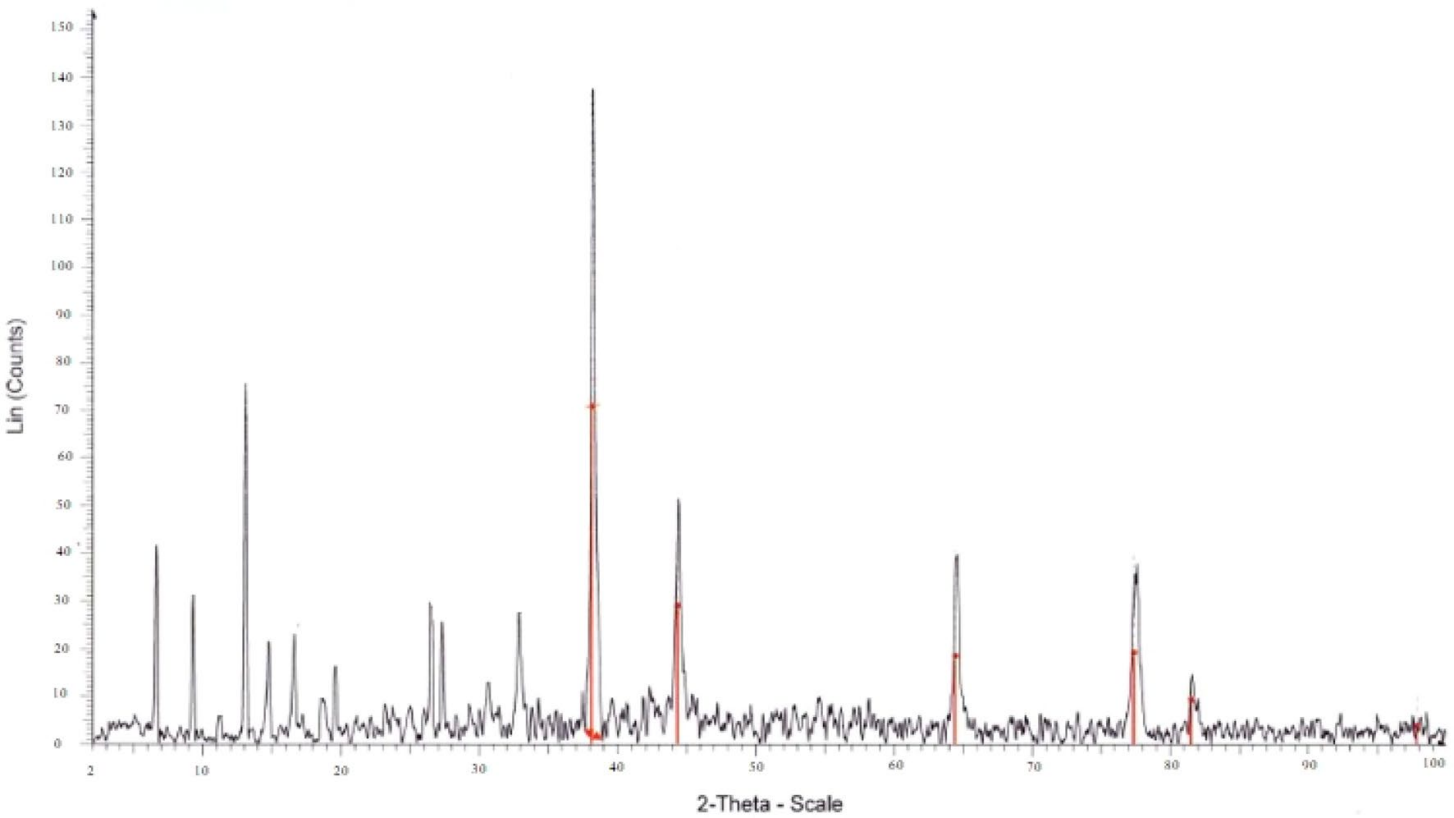
XRD pattern of Co-MOF@Ag_2_O nanocomposite.

X-ray diffraction patterns of annealed samples are depicted in Fig. 4. The XRD patterns reveal the polycrystalline nature of the synthesized Ag_2_O nanoparticles with cubic phase. The observed diffraction peaks correspond to (111), (200), (220), and (311) planes and well match with standard data of face-centered cubic silver (JCPDS Card No. 04-0783)^51^. Figure 3 also shows the XRD pattern of Co-MOF which indicates multiple diffraction peaks, suggesting the poly-crystalline structure of Co-MOF. The crystallite size was estimated by Scherrer’s formula D = Kλ/*β*cosθ.

Where K = 0.9 (constant), λ = 1.54 Å, β is the Full Width at Half Maximum (FWHM) calculated in radians and θ denotes the Bragg’s diffraction angle. The average crystallite size of the Co-MOF@Ag_2_O nanocomposite was 28.2 nm with excellent dispersity.

**Figure 5:**
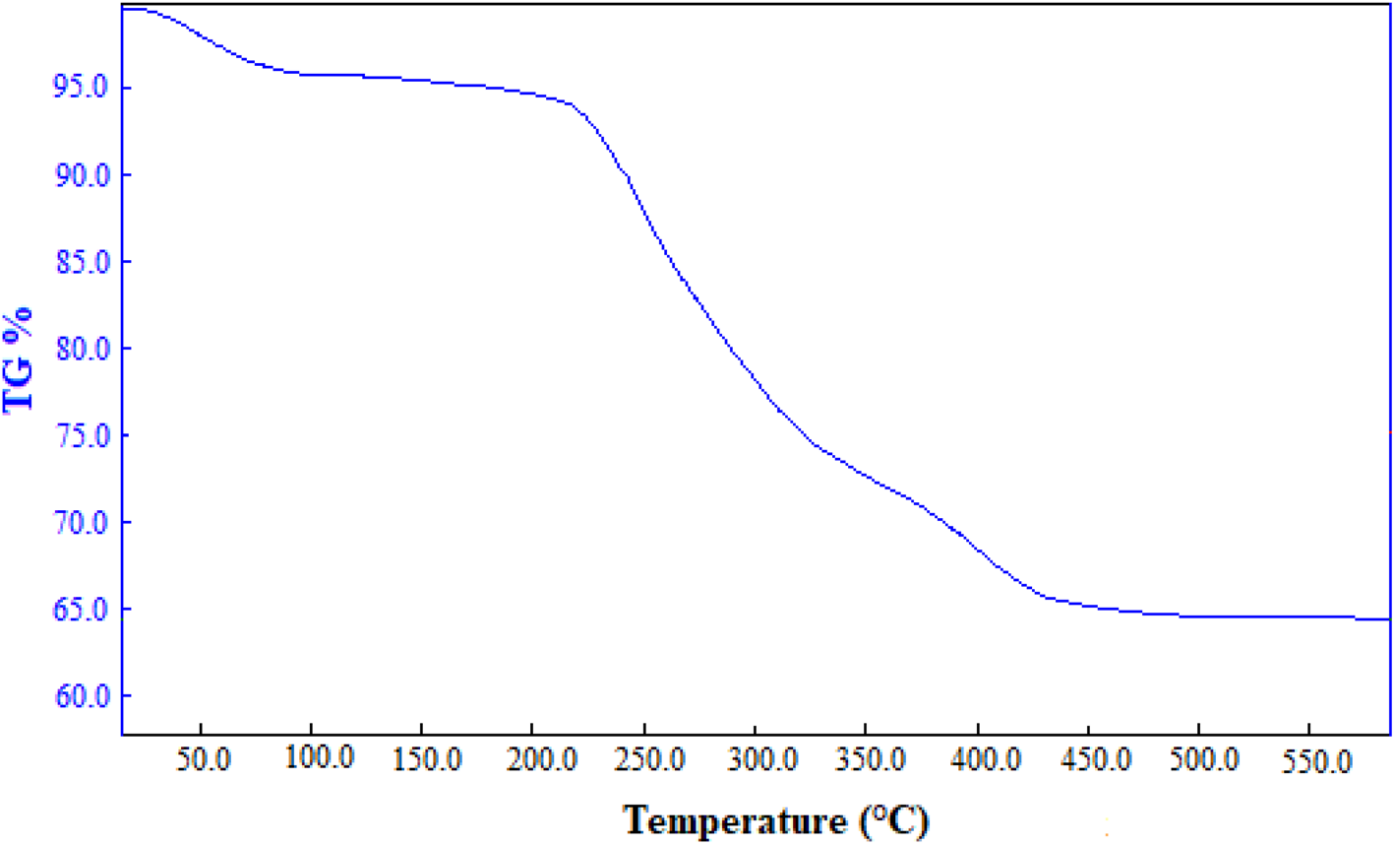
TGA analysis of Co-MOF@Ag_2_O nanocomposite.

The thermogravimetric analysis (TGA) indicates a continuous mass loss of Co-MOF@Ag_2_O nanocomposite before ca. 420 °C (Fig. 5). The weight loss around 100 °C (weight loss: 6.51%) can be related to the evaporation of solvent, while the weight loss between 241 and 373 °C (weight loss: 26.43%) is due to the decomposition of the ligand. The weight loss in the range of 375–478 °C (weight loss: 7.23%), may be attributed to the decomposition of coordinated water. The relatively high thermal decomposition temperature shows that Co-MOF@Ag_2_O nanocomposite has good thermal stability properties.

**Figure 6:**
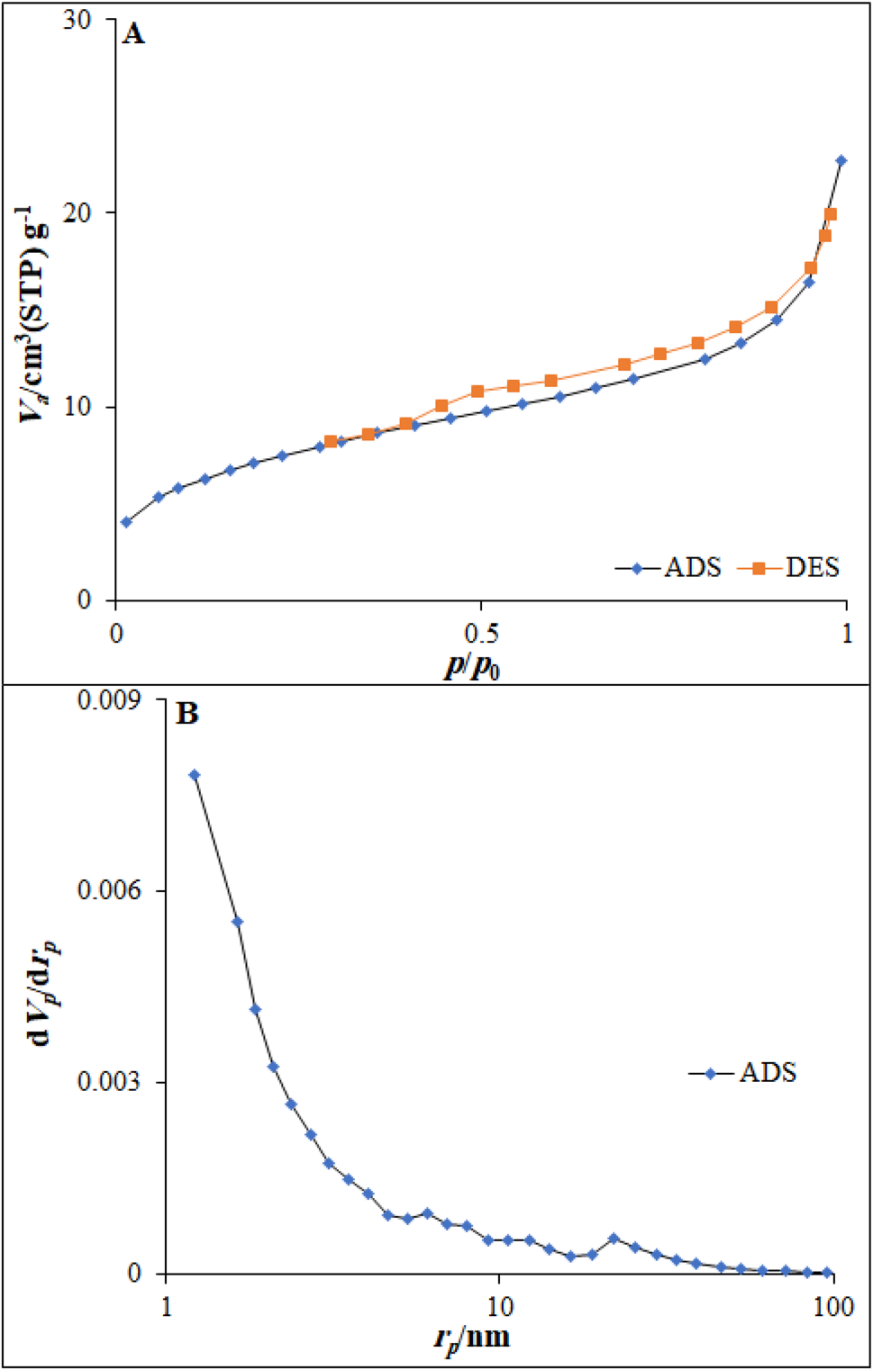
(**A**) N_2_ adsorption–desorption isotherms Co-MOF@Ag_2_O nanocomposite and (**B**) BJH results obtained for Co-MOF@Ag_2_O nanocomposite.

The porous characteristics of the Co-MOF@Ag_2_O nanocomposite were investigated by isothermal nitrogen adsorption–desorption measurements. Figure 6A depicts a typical IV isotherm with a distinct hysteresis loop in the range of 0.39–1.0 *pp*_*0*_^–1^, suggesting the mesoporous structure of the nanocomposite. The pore size distribution was calculated by desorption isotherm via the Barret–Joyner–Halenda (BJH) method as shown in Fig. 6B. The as-synthesized Co-MOF@Ag_2_O nanocomposite exhibited narrow pore-size distribution centered at ~ 5.38 nm, which further confirm the mesoporous structure of the nanocomposite. The Brunauer–Emmett–Teller (BET) specific surface area and pore volume of the nanocomposite were 81.613 m^2^ g^−1^ and 0.12 cm^3^ g^−1^, respectively. The formation of the mesopores was confirmed by the gas release during the decomposition of the precursor.

**Figure 7:**
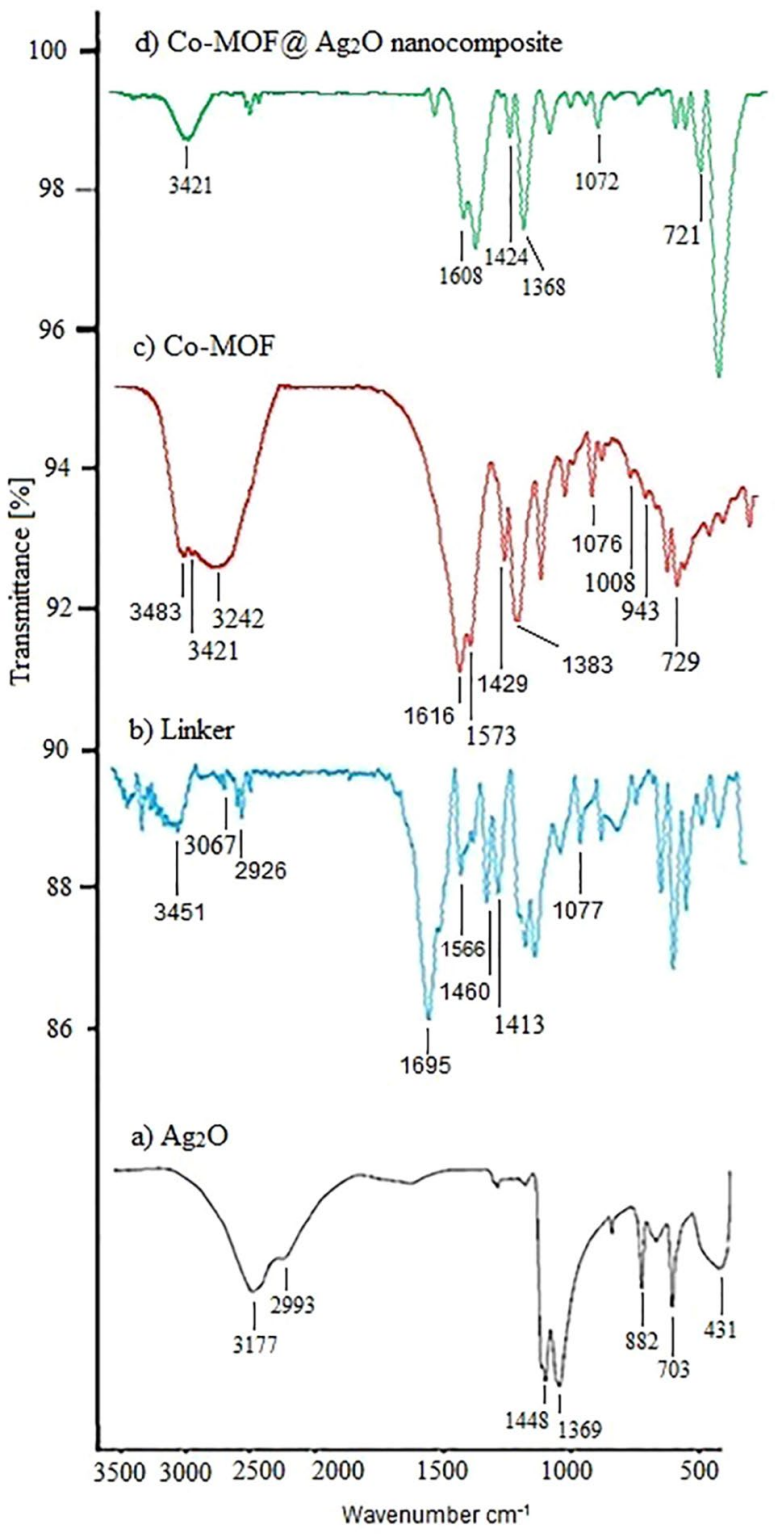
IR (KBr, υ/cm^−1^) curve of (**a**) Ag_2_O, (**b**) 2,6-pyridinedicarboxylic acid linker, (**c**) synthesized Co-MOF and (**d**) Co-MOF@Ag_2_O nanocomposite.

The FT-IR spectra of Ag_2_O, 2,6-pyridinedicarboxylic acid linker, Co-MOF, and Co-MOF@Ag_2_O are shown in Fig. 7. In the IR spectrum of Ag_2_O NPs (7a), the peaks at 703 and 882 cm^−1^ can be attributed to the vibrations of Ag–O whereas the wide band at 431 cm^−1^ can be ascribed to Ag metal.

The FTIR spectrum of 2,6-pyridinedicarboxylic acid linker (7b) shows peaks at 3451, 1413, and 1460 cm^−1^ are related to the stretching vibrations of ∂(OH), C-O, and C-N groups, respectively. The symmetric and asymmetric stretching vibrations of aromatic carboxylates also appeared at 1413–1695 cm^−1^. The peak at 3067 cm^−1^ suggests the presence of free ligand resulting from protonation of the uncoordinated pyridinium ion in the former, and strong hydrogen bonding in the latter^65^.

According to the spectrum of (7c), the peaks emerging at 3483, 3421, 3242, 1573, and 1429 cm^−1^ and those at 1076 cm^−1^ confirmed the presence of the coordinated water, carboxyl group, stretching vibration of C–H, COO group, stretching vibration of C–O bonds, and stretching vibration of (C–C) in the Co–MOF structure, respectively. The peaks at 1008 cm^−1^ can be also assigned to Co–CH and Co in the final Co–MOF nanostructures. The band at ~ 729–943 cm^−1^ can be attributed to the Co–O in the final MOF nanostructures^66^.

One of the signs of the formation of the Co–MOF with the addition of cobalt nitrate to the linker is the reduction of carbonyl absorption due to the coordination of the cobalt metal with the oxygen of the carbonyl groups of the linker.

In the IR spectrum of (7d), a peak at 721 cm^−1^ indicates the presence of Ag_2_O NPs in the final composite. Moreover, the absorption of carbonyl showed a decrease due to the coordination of the Ag_2_O nanoparticle with the oxygen of the carbonyl group. These two signs confirm the presence of silver nanoparticles in the Co-MOF structure and its contribution to the formation of the Co-MOF@Ag_2_O complex.

### Synthesis of dihydropyrazolopyranopyrimidine diones in the presence of Co-MOF@Ag_2_O as nano-organocatalyst

**Figure 8:**
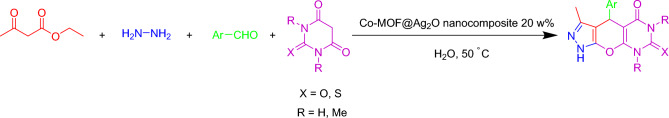
Four-component synthesis of pyrazolopyranopyrimidines catalyzed by Co-MOF@Ag_2_O in water at 50 °C.

As shown in Fig. 8, the optimum reaction conditions for the synthesis of pyrazolopyranopyrimidine in the presence of Co-MOF@A_g2_O catalyst involves the one-pot four-component reaction of ethyl acetoacetate, hydrazine hydrate, 3-nitro benzaldehyde, and barbituric acid.

First, the impacts of the catalyst loading were examined within the model reaction at 50 ºC and water as the medium. In such conditions, 20 wt% of the catalyst was the optimal content to complete the reaction with higher efficiency and shorter reaction time (Table 1, entry 5). No effects were reported on the product efficiency when the catalyst amount was increased (Table 1, entries 6, 7), while the desired product showed a lower yield when lower percentages of Co-MOF@Ag_2_O were employed (Table 1, entries 2, 3, and 4). The four-component reaction was conducted in water at 50 °C with no catalytic agent to find the actual effectiveness of the catalyst. The results of Table 1 (entry 1) indicated the achievement of the trace product even after 2 h of reaction.

**Table 1:** Optimization of the reaction conditions for the synthesis of pyrazolopyranopyrimidines using Co-MOF@Ag_2_O nanocomposite.

Entry^a^	Catalyst	Solvent	Tem (°C)	Time (min)	Yield^b^ (%)
1	–	H_2_O	50 °C	120	Trace
2	Co-MOF@Ag_2_O 5%	H_2_O	50 °C	10	75
3	Co-MOF@Ag_2_O 10%	H_2_O	50 °C	10	80
4	Co-MOF@Ag_2_O 15%	H_2_O	50 °C	4	83
5	Co-MOF@Ag_2_O 20%	H_2_O	50 °C	3	96
6	Co-MOF@Ag_2_O 25%	H_2_O	50 °C	3	96
7	Co-MOF@Ag_2_O 30%	H_2_O	50 °C	3	97
8	Co-MOF@Ag_2_O 20%	CH_3_CN	50 °C	57	67
9	Co-MOF@Ag_2_O 20%	DMF	50 °C	45	72
10	Co-MOF@Ag_2_O 20%	MeOH: H_2_O	50 °C	14	79
11	Co-MOF@Ag_2_O 20%	EtOH: H_2_O	50 °C	10	88
12	Co-MOF@Ag_2_O 20%	EtOH	50 °C	25	79
13	Co-MOF@Ag_2_O 20%	MeOH	50 °C	45	71
14	Co-MOF@Ag_2_O 20%	Solvent free	50 °C	12	63
15	Co-MOF@Ag_2_O 20%	H_2_O	r.t.	145	Trace
16	Co-MOF@Ag_2_O 20%	H_2_O	70 °C	2	98

^a^Reaction conditions: 3-nitrobenzaldehyde (1 mmol), barbituric acid (1 mmol), ethyl acetoacetate (1 mmol), hydrazine hydrate (1 mmol) in presence of Co-MOF@Ag_2_O nanocomposite and solvent (10 mL).

^b^Yield refers to isolated products.

Table 1 also indicates temperature effects on the conversion, revealing a low product yield at room temperature (Table 1, entry 15). The product yield improved significantly by raising the temperature from room temperature to 50 °C. However, as shown in Table 1 (entry 16), further temperature elevation from 50 to 70 °C caused no changes in the product yield and reaction time.

Different solvents, including polar aprotic (DMF, CH_3_CN) and polar protic (H_2_O, MeOH, EtOH, EtOH–water, and MeOH–water mixture) were used to conduct the model reaction and examine the effects of the solvent. Water showed the highest effectiveness as the solvent, leading to the smooth proceeding of the four-component reaction at the greatest yield. Significant changes were observed in the reaction profile by changing to the aprotic polar solvent acetonitrile, prolonging the reaction. The use of protic solvents affected the reaction positively through hydrogen bonding with the substrates. Besides, the hydrophobic effects of water may justify the superiority of water compared to ethanol. Another point is the low yield of the target product when performing the reaction in solvent-free conditions (Table 1, entry 14), confirming the high reliance of the reaction on the solvent.

According to Table 2, different barbituric acids and aldehydes with electron-donating and withdrawing groups were used to examine the scope and generality of the proposed technique by performing the reaction at optimum conditions. As shown, excellent yields were obtained by all benzaldehyde derivatives. Yet, slightly greater yields were achieved in the case of the electron-withdrawing group. Moreover, an 84% product yield was reported when three electron-donating methoxy groups were present on the aromatic ring of the aldehyde (Table 2, entry 18). On the other hand, the yield of the reaction with barbituric acid was slightly higher than thiobarbituric acid and N, N-dimethyl barbituric acid because of less sterically challenging barbituric acid. The product 5r was characterized in terms of melting point and spectral data, such as FT-IR and NMR spectra.

**Table 2:** Preparation of pyrazolopyranopyrimidines in the presence of Co-MOF@Ag_2_O (20 w%) under optimized reaction conditions.

Entry^a^	Aldehyde	R	X	Product	Time (min)	Yield (%)^b^	m.p. (°C)	
							Found	Reported [ref.]
1	3-NO_2_C_6_H_4_^−^	H	O	5a	10	92	268–270	266–267^54^
2	2-NO_2_C_6_H_4_^−^	H	O	5b	6	90	208–210	208–209^54^
3	4-NO_2_C_6_H_4_^−^	H	O	5c	4	92	231–233	233–234^54^
4	C_6_H_5_^−^	H	O	5d	6	96	219–220	218–219^54^
5	C_6_H_5_^−^	H	S	5e	5	94	222–223	220–221^54^
6	C_6_H_5_^−^	Me	O	5f.	5	93	194–195	192–193^54^
7	4-ClC_6_H_4_^−^	H	O	5 g	11	98	222–223	222–223^54^
8	4-ClC_6_H_4_^−^	Me	O	5 h	13	94	199–201	199–200^54^
9	4-ClC_6_H_4_^−^	H	S	5i	9	95	231–233	232–239^67^
10	4-BrC_6_H_4_^−^	H	O	5j	5	96	210–211	211–212^67^
11	2-CH_3_C_6_H_4_^−^	H	O	5 k	8	92	261–264	260–262^68^
12	4-CH_3_C_6_H_4_^−^	H	O	5 l	7	94	198–201	200–201^54^
13	4-CH_3_C_6_H_4_^−^	Me	O	5 m	10	89	172–174	172–173^54^
14	4-CH_3_C_6_H_4_^−^	H	S	5n	9	89	219–221	219^59^
15	4-OCH_3_C_6_H_4_^−^	H	O	5o	6	91	227–229	225–227^69^
16	4-OCH_3_C_6_H_4_^−^	H	S	5p	8	88	222–223	224–226^70^
17	4-OCH_3_C_6_H_4_^−^	Me	O	5q	10	86	175–177	175–177^70^
18	3,4,5-(OCH_3_)_3_C_6_H_2_^−^	H	O	5r	12	84	248–249	248–250^68^
19	2-OHC_6_H_4_^−^	H	O	5 s	5	94	263–265	264–266^68^
20	4-OHC_6_H_4_^−^	H	O	5q	5	95	263–267	262–264^68^
21	2-OH-1-naphthaldehyde	H	O	5r	14	90	220–221	–

^a^Reaction conditions: Aryl aldehydes (1 mmol), barbituric acid derivatives (1 mmol), ethyl acetoacetate (1 mmol) hydrazine hydrate (1 mmol) in the presence of Co-MOF@Ag_2_O were heated in H_2_O at 50 °C for appropriate times.

^b^Yield refers to isolated products.

**Figure 9:**
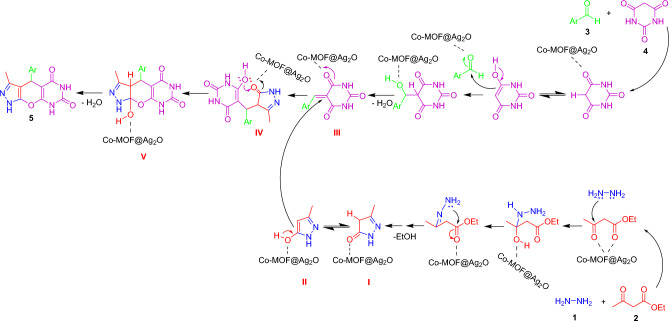
The proposed mechanism for synthesis of pyrazolopyranopyrimidines.

Figure 9 shows the proposed Co-MOF@Ag_2_O-catalyzed mechanisms to synthesize pyrazolopyranopyrimidine derivatives from ethyl acetoacetate, hydrazine hydrate, benzaldehyde, and barbituric acid. The process represented a typical Knoevenagel condensation cascade followed by Michael addition and cyclization Co-MOF@Ag_2_O. Initially, the carbonyl group in ethyl acetoacetate (2) that activated by catalyst was attacked by hydrazine hydrate (1).

The resulting adduct condensation was converted to intermediate I as it lost a molecule of water; then, EtOH was tautomerized to its relative equilibrium enolate form II in the presence of Co-MOF@Ag_2_O. Yet, Knoevenagel condensation of activated aldehyde and barbituric acid in the presence of Co-MOF@Ag_2_O led to the formation of intermediate III. Then, the reaction of intermediate (II) occurs with the Knoevenagel condensate (III) product through a Michael addition reaction to produce intermediate (IV). The nucleophilic addition of the enolate oxygen to the activated carbonyl group leads to the intramolecular cyclization of intermediate (IV) and afforded intermediate (V). In the last step, the Co-MOF@Ag_2_O catalyst assists intermediate V to lose a water molecule and form target product 5.

Table 3 summarizes the previous procedures used to synthesize fused tricyclic pyrazolopyranopyrimidine derivatives, highlighting the value of the proposed catalyst compared to others. For this purpose, preparation of 3-methyl-4-phenyl-4,8-dihydropyrazolo[4′,3′:5,6]pyrano[2,3-d]pyrimidine-5,7(1H,6H)-dione (5d) using Co-MOF@Ag_2_O was compared with other reported methods. As illustrated, some previously used procedures required higher temperatures, longer reaction times, and challenging processes to prepare the catalyst at proper product yield.

**Table 3:** The comparison of the catalytic activity of Co-MOF@Ag_2_O with previously reported catalysts.

Entry	Catalyst	Amount of catalyst	Conditions	Time (min)	Yield (%)	Ref.
1	[BNPs-Caff] HSO_4_	0.1 g	H_2_O, 50 °C	40	95	^71^
2	MNPs@DABCO^+^Cl^-^	0.01 g	Solvent free, 80 °C	5	94	^72^
3	DABCO	20 mol%	H_2_O, Reflux	20	99	^48^
4	Meglumine	0.1 mmol	H_2_O, r.t	5	97	^54^
5	[MerDABCO-SO_3_H] Cl	5 mg	H_2_O, 80 °C	5	97	^73^
6	HPA-F-HNTs	0.03 g	H_2_O, Reflux	35	96	^50^
7	TEDA/IMIZ-BAIL@UiO-66	0.05 g	EtOH, Reflux	40	93	^74^
8	Cu^2+^@MSNs-(CO^2 –^)_2_	1.3 mol%	H_2_O, r.t.	60	90	^61^
9	Oleic acid	12.5 mol%	EtOH, Reflux	15	78	^60^
10	TiO_2_ NWs	10 mol%	EtOH: H_2_O, reflux	60	95	^53^
11	SBA-Pr-SO_3_H	0.02 g	H_2_O, Reflux	10	92	^59^
12	CO-MOF@Ag_2_O	20 w%	H_2_O, 50 °C	10	92	This work

One of the best results was reported for Co-MOF@Ag_2_O in water as a catalyst, being operationally simple and recyclable, requiring a shorter reaction time, leading to excellent yields, and a wide substrate range with high functional group tolerance. The two features of cost-effectiveness and the use of water as a green solvent show the superiority of this catalyst over previous catalysts.

**Figure 10:**
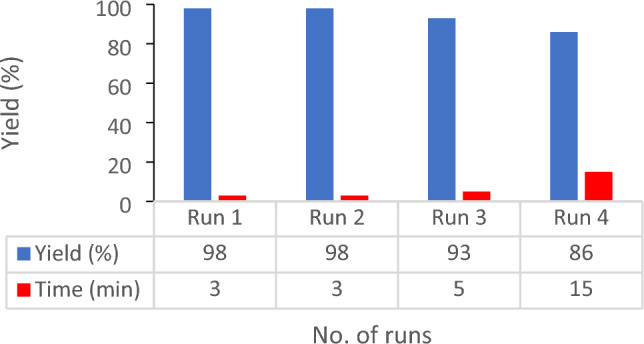
Reusability of catalyst.

The reusability and stability of Co-MOF@Ag_2_O nanocatalysts have an important role in determining the economic feasibility of pyrazolopyranopyrimidines production at a large industrial scale. Therefore, the reusability of the Co-MOF@Ag_2_O nano-catalyst was investigated for the reaction of 3-nitrobenzaldehyde, barbituric acid, ethyl acetoacetate and hydrazine hydrate as model reaction to produce 5a. After completion of the reaction (monitored by TLC), with separating the obtained precipitates by filtration and dissolving them in ethanol, the Co-MOF@Ag_2_O nano-catalyst was separated, washed with water and ethanol (5 mL) and dried in oven at 65 °C for 6 h. It was used in the next reaction. The results depicted that Co-MOF@Ag_2_O nano-catalyst can be reused up to 4 times. After being used more than 4 times, Co-MOF@Ag_2_O nano-catalyst lost activity gradually (Fig. 10).

**Figure 11:**
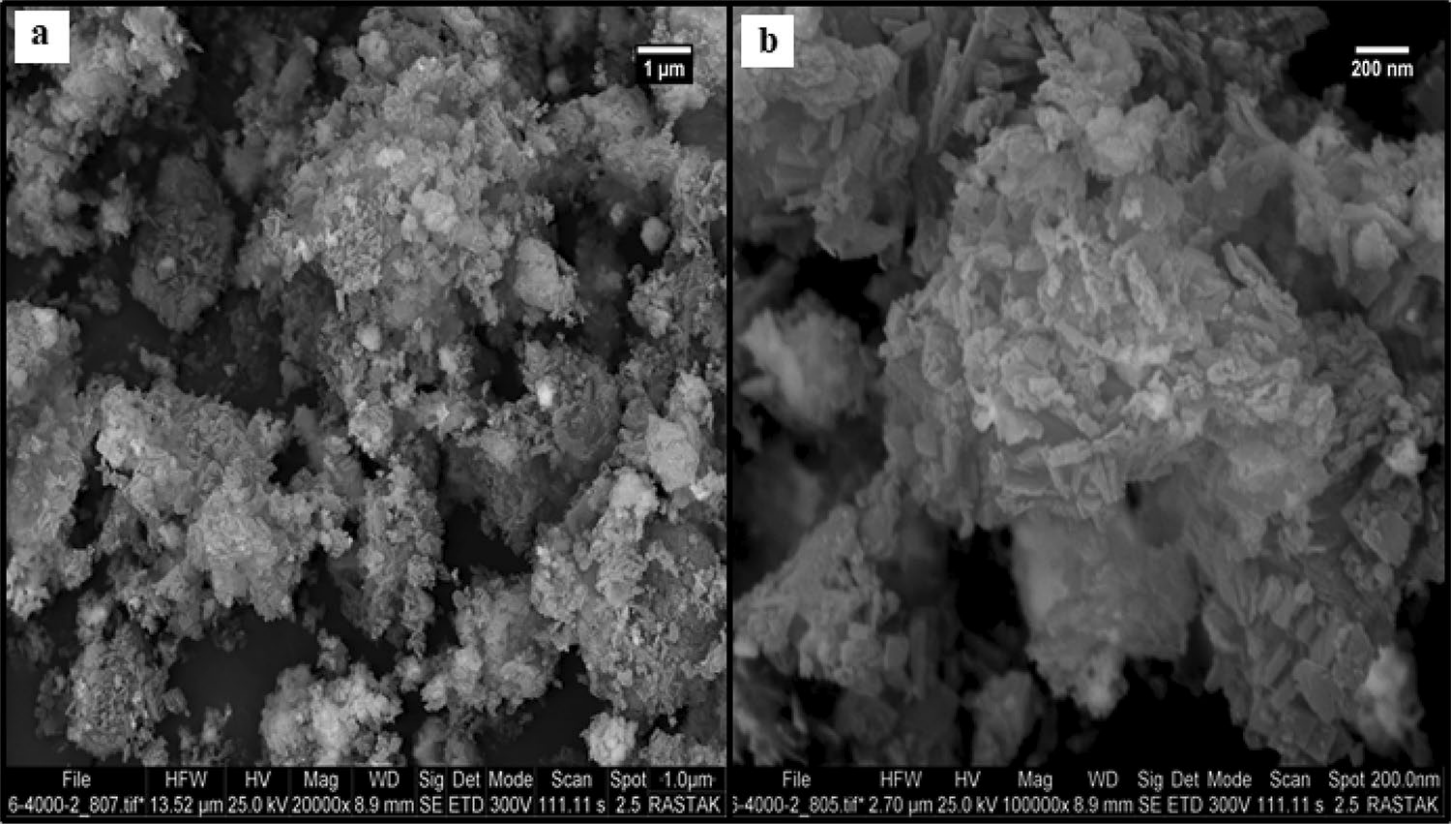
(**a**) FE-SEM image and (**b**) high-resolution FE-SEM image of Co-MOF@Ag_2_O nano-catalyst showing change on the catalyst surface structure during the last run (4th) reuse cycle.

The loss of catalytic activity of nano-catalysts may be ascribed to its structural changes. The FE-SEM micrograph of the nano-catalyst (Fig. 11) confirmed the stable surface for the products.

**Figure 12:**
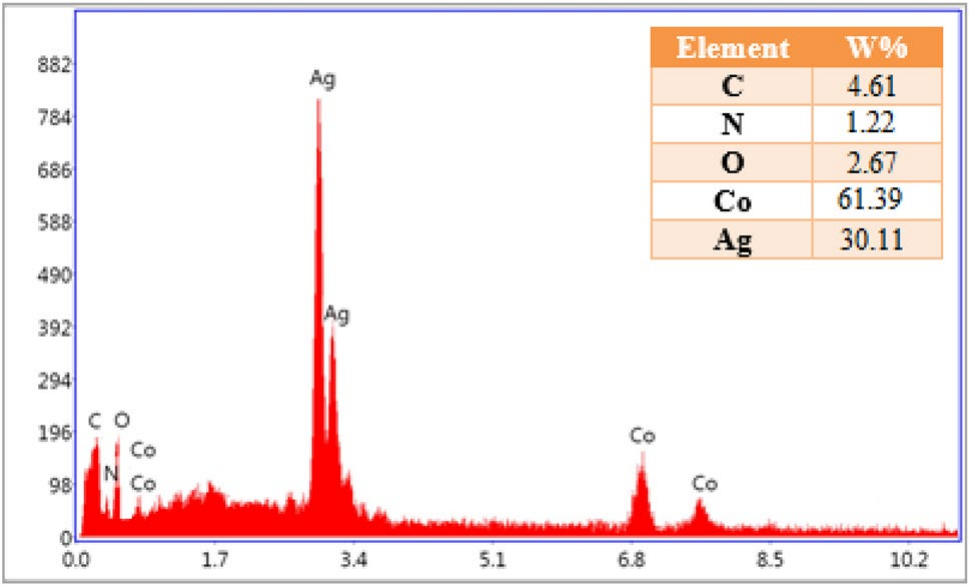
EDX spectra of Co-MOF@Ag_2_O nano-catalyst during the last run (4th) reuse cycle.

Furthermore, we have characterized the reused nano-catalyst by EDX and FT-IR analyses which EDX analysis showed the presence of carbon, nitrogen, oxygen, cobalt, and silver elements in the reused nano-catalyst. This analysis result is shown in Fig. 12.

**Figure 13:**
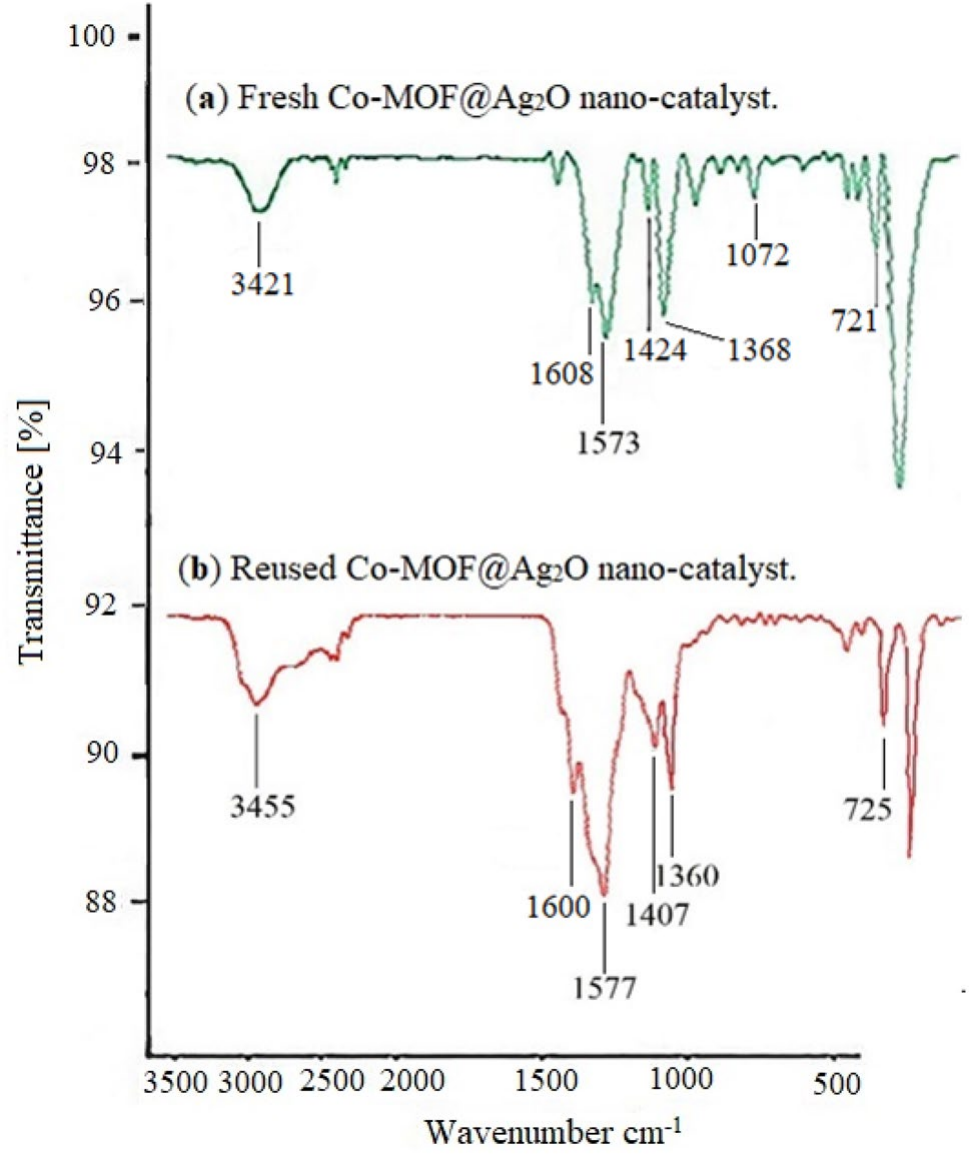
(**a**) FT-IR spectra of fresh Co-MOF@Ag_2_O nano-catalyst and (**b**) FT-IR spectra of Co-MOF@Ag_2_O nano-catalyst during the last run (4th) reuse cycle.

In FT-IR spectrum Fig. 13, The existence of a similar pattern including the absorptions of 3421 and 3455 cm^−1^ related to the hydroxyl groups in the Co-MOF@Ag_2_O nano-catalyst, the absorptions of 721 and 725 cm^−1^ related to Ag–O vibrations in Ag_2_O NPs, the absorptions of the carbonyl groups in linker at 1608 and 1600 cm^−1^ and the absorptions of 1368–1573 cm^−1^ and 1360–1577 cm^−1^ related to the aromatic ring, confirming no change in the structure of the Co-MOF@Ag_2_O nano-catalyst.

To approve the heterogeneous nature of the Co-MOF@Ag_2_O nanocomposite and the chances of leaching of metal cites of catalyst in the reaction mixture a hot filtration test was performed for the synthesis of pyrazolopyranopyrimidines using 3-nitrobenzaldehyde, barbituric acid, ethyl acetoacetate and hydrazine hydrate as model reaction to produce 5a under the optimized reaction condition. After half time of the reaction (5 min), the reaction was stopped, and the corresponding product was obtained in 71% of yield. The Co-MOF@Ag_2_O nanocomposite was separated and removed from the reaction by dissolving precipitates in ethanol and simple filtration. After evaporating the ethanol from the reaction residue, the rest of the reaction was stirred in the catalyst-free conditions for another 5 min. Surprisingly, we observed that low conversion (< 5%) of the product happened through the heating of the catalyst-free mixture for another 5 min which may be a result of the thermal energy applied. It can be concluded that the catalyst has high stability, no active metal centers were leached from the nanocomposite surface.

## Conclusion

In conclusion, a nanocatalyst was constructed using Ag_2_O nanoparticles which stabilized on the surface of Co-MOF using microwave irradiation and was applied after identification and characterization by SEM, EDX, XRD, TGA, BET, and FT-IR analysis for the preparation of pyrazolopyranopyrimidine heterocycles through a one-pot four-component reaction of ethyl acetoacetate, hydrazine hydrate, aryl aldehydes, and barbituric acid derivatives under solvent-free conditions at 50 °C. The synthesized nano-catalyst in this work indicates more catalytic activity in comparison with the previously proposed procedures. The convenient preparation process, catalyst recyclability, considerable atom economy, and solvent-free and green reaction conditions are among the main strengths of the proposed protocol in this research.

### Supplementary Information

Supplementary Information.

## Data Availability

The original contributions presented in the study are included in the article/Supplementary material; further inquiries can be directed to the corresponding author.
