# Supplementary material for: Synthesis and characterization of Co-MOF@Ag2O nanocomposite and its application as a nano-organic catalyst for one-pot synthesis of pyrazolopyranopyrimidines
Source: Sci Rep. 2023 Oct 15;13:17500. doi: 10.1038/s41598-023-44667-6 (PMC10577138; doi:10.1038/s41598-023-44667-6)
Supplement: Supplementary file 1 — Supplementary Information. [file 41598_2023_44667_MOESM1_ESM.docx]

***Supplementary Material***

**Synthesis and characterization of** [**Co-MOF@Ag_2_O**](mailto:Fe-MOF@Fe3O4) **nanocomposite and its application as a** **nano-organic catalyst for one-pot synthesis of pyrazolopyranopyrimidines**

**Ghader Hootifard ^1^, Enayatollah Sheikhhosseini ^1^*, Sayed Ali Ahmadi ^1^, Mahdieh Yahyazadehfar ^1^**

**^1^ Department of Chemistry, Kerman Branch, Islamic Azad University, Kerman, Iran**

*** Correspondence:**

Enayatollah Sheikhhosseini
sheikhhosseiny@gmail.com or sheikhhosseini@iauk.ac.ir

**^1^H NMR and ^13^C NMR of compound (5r)**

*4-(2-hydroxynaphthalen-1-yl)-3-methyl-4,8-dihydropyrazolo[4',3':5,6]pyrano[2,3-d]pyrimidine-5,7(1H,6H)-dione (****5r****)*: Yield 90 %, m.p: 220-221 °C; ^1^H NMR (400 MHz, DMSO-*d_6_,* ppm): δ 2.62 (s, 3H, CH_3_), 5.35 (s, 1H, CH), 7.38 (d, 1H, *J*_1_ = 9.2 Hz), 7.51-7.55 (m, 2H, 1H-Ar, 1H-NH), 7.62 (td, 1H, *J*_1_ = 15.6 Hz, *J*_2_ = 1.2 Hz, H-Ar), 7.70 (td, 1H, *J*_1_ = 15.6 Hz, *J*_2_ = 1.2 Hz, H-Ar), 7.97 (d, *J* = 8.8 Hz, 1H, H-Ar), 8.11 (d, *J* = 9.2 Hz, 1H, H-Ar), 8.74 (s, 1H, OH), 10.0 (s, 1H, NH), 12.98 (s, 1H, NH) ppm; ^13^C NMR (100 Hz, DMSO-*d*_6_): δ 10.01, 23.3, 87.9, 108.4, 116.4, 118.9, 121.8, 123.8, 124.9, 127.0, 128.0, 129.0, 130.7, 131.0, 132.3, 134.7, 160.0, 161.3 ppm.

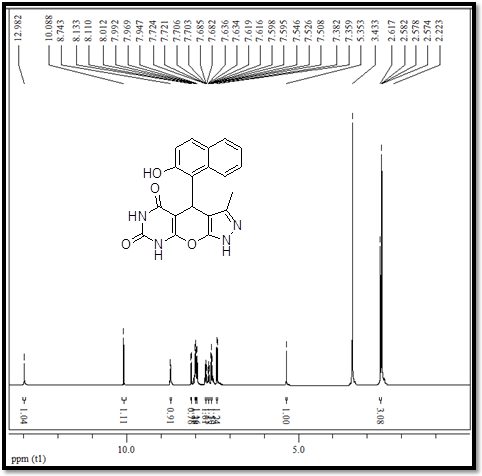


**Figure S-1.** ^[1](https://www.google.com/url?sa=t&rct=j&q=&esrc=s&source=web&cd=&cad=rja&uact=8&ved=2ahUKEwjmjuPvw579AhW_8LsIHTvtAd8QFnoECBAQAQ&url=https%3A%2F%2Fwww.researchgate.net%2Ffigure%2FThe-1-H-NMR-spectrum-of-compound-3a-in-CDCl-3-solvent_fig1_320865085&usg=AOvVaw2S68h8geITZVHy7tcJjbIy)^[H NMR spectrum of compound 5r.](https://www.google.com/url?sa=t&rct=j&q=&esrc=s&source=web&cd=&cad=rja&uact=8&ved=2ahUKEwjmjuPvw579AhW_8LsIHTvtAd8QFnoECBAQAQ&url=https%3A%2F%2Fwww.researchgate.net%2Ffigure%2FThe-1-H-NMR-spectrum-of-compound-3a-in-CDCl-3-solvent_fig1_320865085&usg=AOvVaw2S68h8geITZVHy7tcJjbIy)


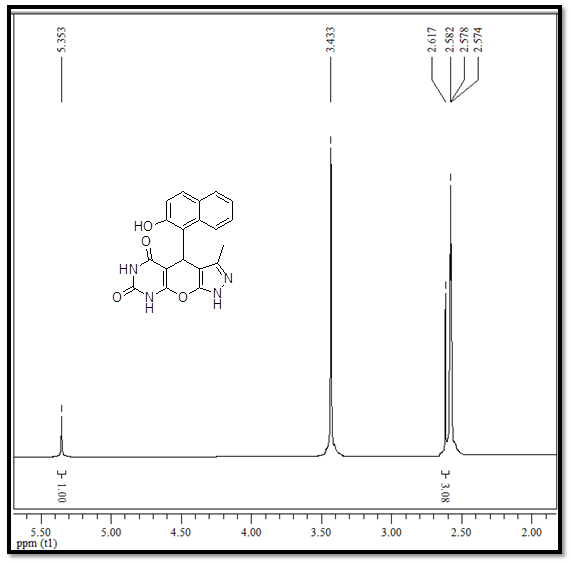


**Figure S-1a.**^[1](https://www.google.com/url?sa=t&rct=j&q=&esrc=s&source=web&cd=&cad=rja&uact=8&ved=2ahUKEwjmjuPvw579AhW_8LsIHTvtAd8QFnoECBAQAQ&url=https%3A%2F%2Fwww.researchgate.net%2Ffigure%2FThe-1-H-NMR-spectrum-of-compound-3a-in-CDCl-3-solvent_fig1_320865085&usg=AOvVaw2S68h8geITZVHy7tcJjbIy)^[H NMR expand spectrum of compound 5r.](https://www.google.com/url?sa=t&rct=j&q=&esrc=s&source=web&cd=&cad=rja&uact=8&ved=2ahUKEwjmjuPvw579AhW_8LsIHTvtAd8QFnoECBAQAQ&url=https%3A%2F%2Fwww.researchgate.net%2Ffigure%2FThe-1-H-NMR-spectrum-of-compound-3a-in-CDCl-3-solvent_fig1_320865085&usg=AOvVaw2S68h8geITZVHy7tcJjbIy)

[5o.](https://www.google.com/url?sa=t&rct=j&q=&esrc=s&source=web&cd=&cad=rja&uact=8&ved=2ahUKEwjmjuPvw579AhW_8LsIHTvtAd8QFnoECBAQAQ&url=https%3A%2F%2Fwww.researchgate.net%2Ffigure%2FThe-1-H-NMR-spectrum-of-compound-3a-in-CDCl-3-solvent_fig1_320865085&usg=AOvVaw2S68h8geITZVHy7tcJjbIy)


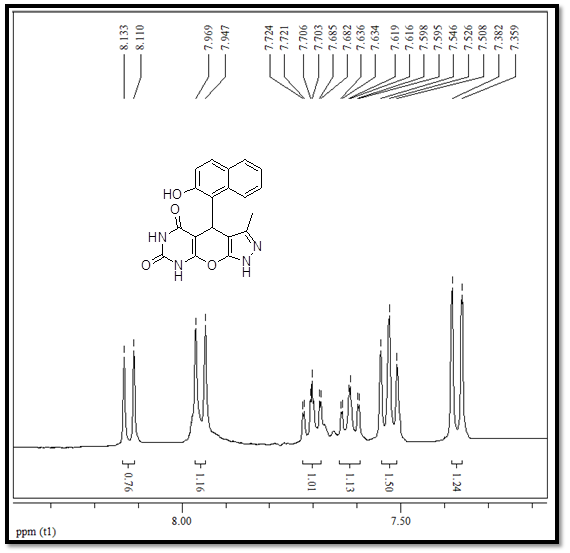


**Figure S-1b.** ^[1](https://www.google.com/url?sa=t&rct=j&q=&esrc=s&source=web&cd=&cad=rja&uact=8&ved=2ahUKEwjmjuPvw579AhW_8LsIHTvtAd8QFnoECBAQAQ&url=https%3A%2F%2Fwww.researchgate.net%2Ffigure%2FThe-1-H-NMR-spectrum-of-compound-3a-in-CDCl-3-solvent_fig1_320865085&usg=AOvVaw2S68h8geITZVHy7tcJjbIy)^[H NMR expand spectrum of compound 5r.](https://www.google.com/url?sa=t&rct=j&q=&esrc=s&source=web&cd=&cad=rja&uact=8&ved=2ahUKEwjmjuPvw579AhW_8LsIHTvtAd8QFnoECBAQAQ&url=https%3A%2F%2Fwww.researchgate.net%2Ffigure%2FThe-1-H-NMR-spectrum-of-compound-3a-in-CDCl-3-solvent_fig1_320865085&usg=AOvVaw2S68h8geITZVHy7tcJjbIy)


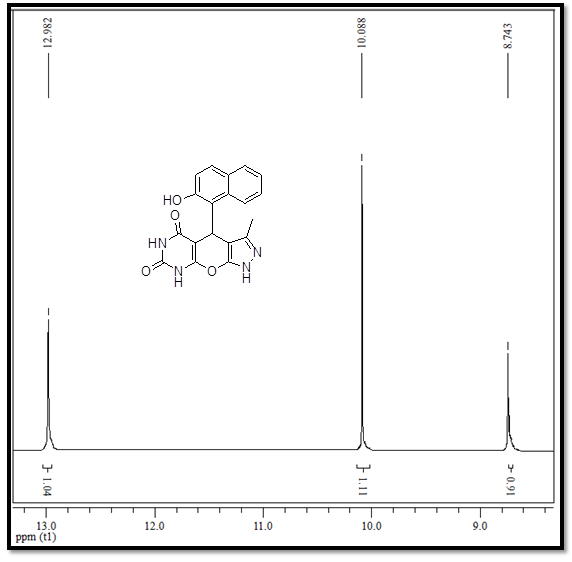


**Figure S-1c.** ^[1](https://www.google.com/url?sa=t&rct=j&q=&esrc=s&source=web&cd=&cad=rja&uact=8&ved=2ahUKEwjmjuPvw579AhW_8LsIHTvtAd8QFnoECBAQAQ&url=https%3A%2F%2Fwww.researchgate.net%2Ffigure%2FThe-1-H-NMR-spectrum-of-compound-3a-in-CDCl-3-solvent_fig1_320865085&usg=AOvVaw2S68h8geITZVHy7tcJjbIy)^[H NMR expand spectrum of compound 5r.](https://www.google.com/url?sa=t&rct=j&q=&esrc=s&source=web&cd=&cad=rja&uact=8&ved=2ahUKEwjmjuPvw579AhW_8LsIHTvtAd8QFnoECBAQAQ&url=https%3A%2F%2Fwww.researchgate.net%2Ffigure%2FThe-1-H-NMR-spectrum-of-compound-3a-in-CDCl-3-solvent_fig1_320865085&usg=AOvVaw2S68h8geITZVHy7tcJjbIy)


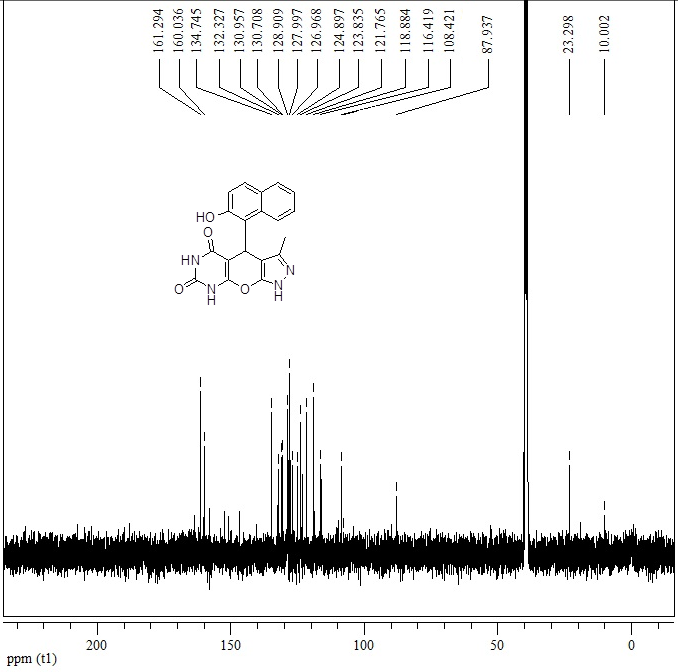


**Figure S-2.** ^13^C NMR spectrum of compound 5r.
